# Development and implementation of a multifunctional mobile robot training kit in embedded control systems instruction in vocational education

**DOI:** 10.1016/j.ohx.2026.e00789

**Published:** 2026-05-11

**Authors:** Herlin Setyawan, Sukardi, Risfendra, Habibullah, Ganefri, Muhammad Anwar

**Affiliations:** a Department of Electrical Engineering, Universitas Negeri Padang, Jl. Prof. Dr. Hamka, Air Tawar Barat, Padang Utara, Padang, Indonesia; b Department of Electronics Engineering, Universitas Negeri Padang, Jl. Prof. Dr. Hamka, Air Tawar Barat, Padang Utara, Padang, Indonesia

**Keywords:** Vocational education, Embedded control systems, Instructional media, Mobile robotics, Teaching and learning

## Abstract

The development of automation, robotics, and embedded intelligence in industry requires that vocational education graduates possess strong competencies in embedded control systems. This article presents the design, open-source documentation (electronic, electrical, mechanical, and assembly), and learning implementation of a multifunctional mobile robot training kit developed to support holistic learning of embedded systems and robotics. The main innovation lies in integrating conceptual and project-based learning within a single modular platform that supports smartphone-controlled robots and line and wall following applications. This device is equipped with structured modules and job sheets covering 14 practical topics, ranging from digital-analog input/output programming to implementation as learning projects. Empirical tests on the second generation showed a significant impact on improving students’ knowledge, practical skills, and problem-solving abilities. The third generation was then implemented for one semester with 118 students. Evaluation using the Technology Acceptance Model, expanded to include self-confidence and intrinsic motivation, showed high acceptance and a positive influence on both constructs. Teacher assessments also showed excellent responses across product functionality, design, technical quality, ergonomics, and safety of use. These findings confirm the potential of training kits as an innovative and relevant learning medium for industry-based vocational education*.*

## Graphical abstract

**Figure:**
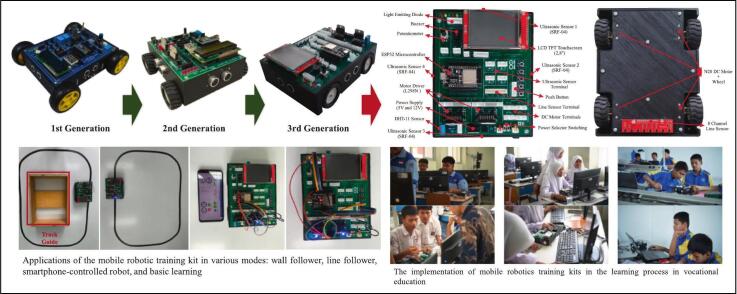

**Table:** 

Hardware name	Training kit mobile robot
Subject area	• Educational tools and open source alternatives to existing infrastructure.
Hardware type	• Robotics • Embedded System
Closest commercial analog	No commercial analog is available.
Open source license	Creative Commons Attribution 4.0 International
Cost of hardware	$80
Source file repository	https://doi.org/10.5281/zenodo.20017448

## 1 Hardware in context

The rapid advancement of industries driven by automation, robotics, the Internet of Things (IoT), mechatronics, and embedded intelligence has established the foundation for smart manufacturing implementation in the modern workplace. These developments have consequently increased the demand for human resources possessing strong competencies in embedded control systems, which serve as the fundamental basis for developing such integrated technologies [1], [2]. Vocational high schools, particularly within the Industrial Electronics Engineering program, play a strategic role in preparing competent human resources for embedded control systems. Graduates of this educational track are expected not only to understand the theoretical concepts of microcontrollers and control systems but also to implement, test, and maintain embedded control systems integrated with sensors, actuators, and mechanical subsystems in real-world applications [3], [4]. Therefore, hardware-based learning has become a crucial element in enhancing vocational students’ competencies to align with the demands of modern manufacturing and automation industries*.*

In an effort to enhance embedded control system competencies among vocational education students, several researchers have implemented mobile robot–based training kits into the learning process, which have been empirically demonstrated to impact students’ competency readiness positively. Through direct interaction with hardware that represents real-world systems, students not only gain deeper conceptual understanding but also develop procedural skills and problem-solving abilities that are essential in industrial electronics [5], [6]. Moreover, several studies have reported significant effects on learning motivation and interest [7], computational thinking skills [8], and students’ mastery of both conceptual and applied knowledge [9]. Instruction in embedded control systems requires a holistic and integrated approach; therefore, the utilization of mobile robot platforms that integrate various components—such as sensors, actuators, and microcontrollers—within a unified learning environment enables students to develop comprehensive competencies encompassing the stages of design, implementation, testing, and evaluation of embedded control systems [5], [9], [10]. Accordingly, the use of mobile robotic training kits enhances students’ cognitive learning outcomes and practical skills. It strengthens the work-readiness of vocational graduates by developing competencies aligned with the demands of modern automation and manufacturing industries.

Several educational tools used in the learning process, such as LEGO Mindstorms [11], [12], Micro: bit [13], [14], mBot [15], and ReFiBot [16], have been shown to have a positive impact on learning in the context of STEM (science, technology, engineering, and mathematics). However, existing tools such as LEGO, Micro: bit, and mBot lack the modularity required for vocational school education because each module already has its own connection terminals, without considering pin configurations or sensor output pin types. Additionally, the programming languages used are still block-based, which differ significantly from the programming language that vocational students must master, namely C. Consequently, the learning approach is more suitable for elementary, middle, or high schools that do not require specific skills and only demand logical, systematic thinking, and problem-solving abilities [11], [12], [13], [14], [15]. Additionally, some researchers have developed the ReFiBot robotics learning platform, which can be used in mobile robotics and also supports C programming; however, the robots designed focus on direct robotics application and do not provide foundational learning to equip students to design systems independently or with teacher guidance [16].

These limitations make existing robotics learning materials unsuitable for vocational education, particularly in embedded control systems. In embedded systems-based electronics education, students are not only required to assemble components but also to accurately understand pin configurations, data types, and the interfacing mechanisms between input–output devices and microcontrollers. This technical literacy is essential because embedded systems education emphasizes the integration of hardware and software, including an understanding of sensors, data communication, and control systems in real-world applications [17], [18]. Additionally, the ability to independently design electronic circuits is a crucial component of the competencies that must be developed, as students are expected not only to use off-the-shelf systems but also to develop microcontroller-based solutions through the design and implementation process [19]. On the other hand, proficiency in low-level programming languages such as C and C++ is a fundamental requirement in the industry, given that these languages are used in embedded system development due to their ability to access hardware directly, their execution efficiency, and their support for real-time systems [5], [19], [20]. Therefore, a learning approach based solely on the use of ready-made robotics platforms tends to be unable to fully develop the in-depth technical competencies required in industry-based embedded systems, which vocational students highly need.

In addition, several previous studies have developed various embedded-control-system learning materials specifically applied to line-following robots [21], wall-following [22], and robots controlled via smartphones [23]. The results of these studies indicate that the use of robotics-based learning media can have a positive impact on vocational education, particularly in enhancing understanding of embedded control system concepts, practical microcontroller programming skills, and the integration of sensors and actuators. However, most developed learning media remain partial and limited to a single function or a specific application scenario. These limitations make such media less flexible for sustained use, whether in foundational learning or during the development and implementation of more complex robotic systems that approximate real-world industrial conditions. Meanwhile, in the learning process, students must undergo foundational learning to grasp concepts and engage in real-world applications to understand the implementation and development of the systems they have studied. Therefore, there is a need to develop modular, multifunctional, and adaptive embedded system learning media that can support holistic learning of embedded control systems without oversimplifying the relevant skills (programming, electronic circuits, and electrical circuits of embedded systems).

Given the limitations of previous studies, the novelty of this research lies in the development of an embedded-system learning medium in the form of a mobile robotics training kit that guides learners from the basics to the application of the system. This training kit is designed to be modular without compromising the pin configuration of each component. Students will independently identify all pin configurations used during the practical exercises. Consequently, students will also design their own electronic systems to create a fully functional embedded control system. It is this modular design that allows the training kit to be used from basic learning to direct application in mobile robotics, as students can select the components used based on the learning being conducted. Consequently, this training kit can be utilized in the learning process by vocational students who lack initial skills and knowledge (those just beginning this course) as well as by students who already possess strong competencies. In practice, three types of robots can be implemented: line-following robots, wall-following robots, and Bluetooth-controlled robots from a smartphone. Consequently, this training kit offers high flexibility in use—it is not limited to project-based lab work but can also be used for foundational understanding. Thus, using this training kit allows students to cover all the learning materials they will study during a full semester of embedded systems instruction.

## 2 Hardware description

**Fig. 1:**
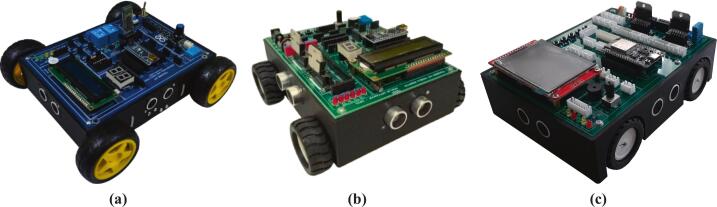
Development of the mobile robotic training kit: (a) gen 1, (b) gen 2, (c) gen 3.

**Fig. 2:**
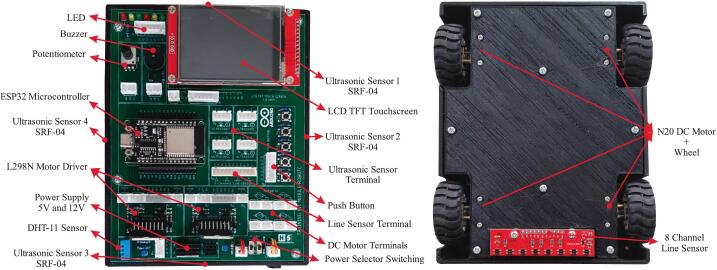
Detailed main components of the third-generation mobile robotic training kit.

The mobile robotic training kit is a microcontroller-based instructional platform utilizing the ESP32, designed to support embedded systems practicum in Industrial Electronics Engineering programs or related fields with similar instructional frameworks. To date, three generations of development and refinement of the mobile robotic training kit have been completed, as illustrated in Fig. 1. This platform is integrated with various input–output components, including ultrasonic sensors, a buzzer, push buttons, a liquid crystal display (LCD), a potentiometer, light-emitting diodes (LEDs), a DC motor driver, line sensors, and a DHT-11 sensor, thereby enabling students to understand the concepts and working principles of hardware interfacing directly. Additionally, it is equipped with DC motors, wheels, and a rechargeable battery, allowing the platform to be used for both autonomous and manual motion experiments, as well as for implementing embedded control algorithms using microcontroller systems. A detailed illustration of the design and types of components used is presented in Fig. 2. Its open design and systematic documentation render this robotic platform relevant not only as a medium for fundamental practicum activities but also as a tool for exploring advanced applications, such as intelligent robotic systems, thereby enhancing students’ conceptual understanding and practical skills in embedded systems engineering. Based on the development outcomes, several key advantages of the mobile robotic training kit can be identified as follows:

• It can be utilized for foundational embedded system instruction, as it integrates various input and output components that can be operated independently.
• It supports real-world embedded system applications in the form of multiple types of mobile robots, including line follower robots, wall follower robots, and smartphone-controlled robots.
• It enables IoT-based applications by employing the ESP32 microcontroller, which supports Wi-Fi and Bluetooth connectivity.
• It can be easily reproduced because it uses readily available materials and cost-effective components.

Although this robotic platform is designed to be reconfigurable—thereby enabling users to adapt it to various applications—this article presents the standard configuration that has been developed. The assembly procedure of the mobile robotic training kit is described in two main sections: electronic assembly and mechanical assembly, which are elaborated in the following subsections. It should be noted that this process excludes the fabrication of the robot’s printed circuit board (PCB), as PCBs are produced through direct industrial manufacturing to ensure high-quality standards*.*

### 2.1 Electronic design

**Fig. 3:**
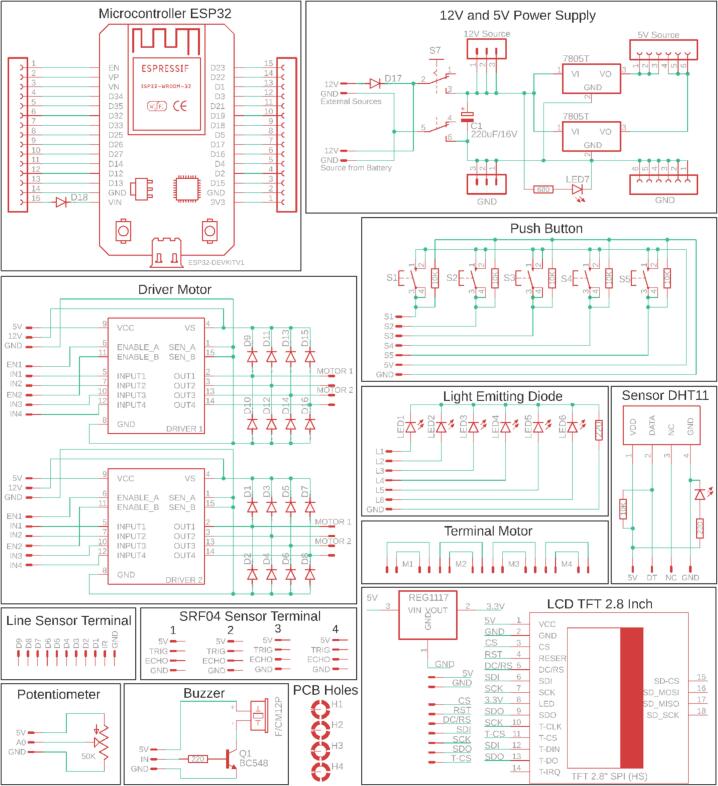
Electronic circuit of the mobile robotic training kit PCB.

**Fig. 4:**
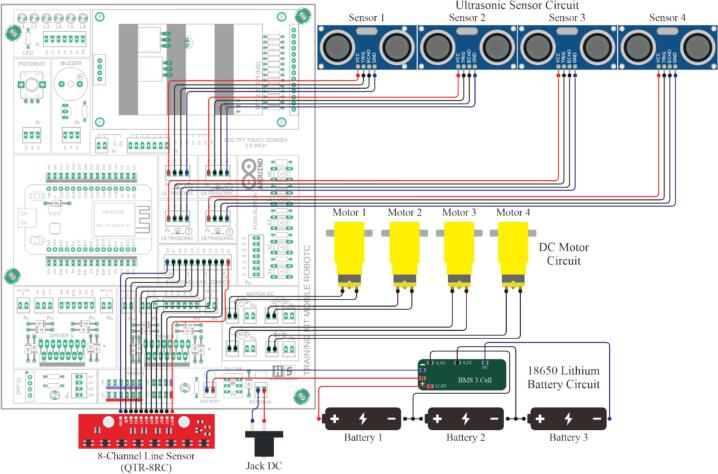
Installation circuit of electronic components separated from the main PCB.

This electronic circuit training kit was created using Eagle software version 9.6.3, and the PCB layout was also designed in this application. The application was used under a student license provided by Autodesk specifically for students for educational purposes [24], [25]. The overall electronic schematic of the mobile robotic training kit is shown in Fig. 3, and the resulting PCB layout and component placement are shown in Fig. 4. As illustrated in Fig. 4, not all components are directly integrated into the main PCB, as their placement follows the structural frame of the training kit. Therefore, the terminals of these components are connected to the designated terminals on the main PCB using cables. Four primary components are physically separated from the main PCB: the SRF04 ultrasonic sensor, the QTR-8RC line sensor, the DC motors, and the battery module. The placement positions of these components are more clearly depicted in Fig. 6, Fig. 7. It is important to note that the ultrasonic sensor includes four main pins: VCC, GND, Trigger, and Echo. Corresponding pins have been provided on the main PCB to allow students to establish proper connections. Therefore, during assembly of the mobile robotic training kit, the pins connecting the sensor to the main PCB must be connected correctly. The same principle applies to all components shown in Fig. 4; careful attention must be given to each pin's function during wiring. This connection process is clearly illustrated in Fig. 4.

**Fig. 5:**
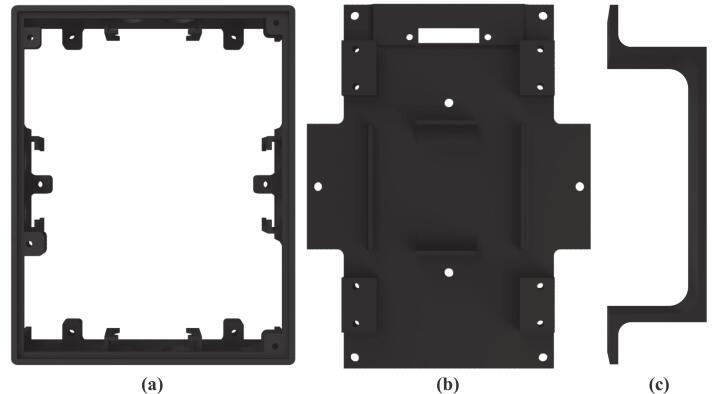
Mechanical design: (a) main robot body, (b) robot chassis, (c) battery cover.

**Fig. 6:**
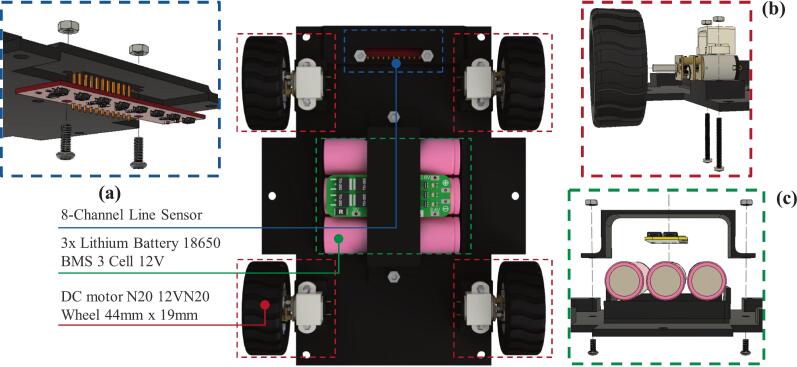
Installation of components on the robot chassis: (a) line sensor installation, (b) motor and wheel installation, (c) battery installation.

**Fig. 7:**
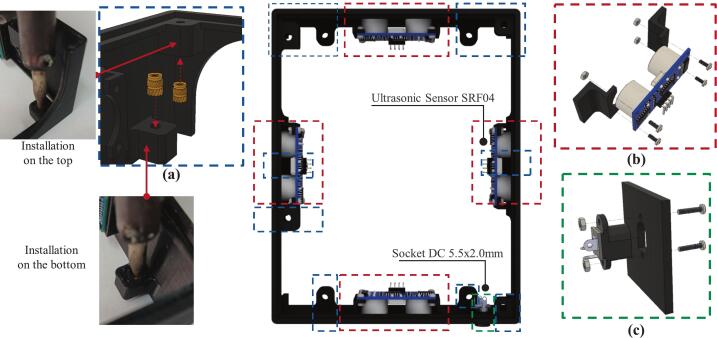
Installation of components on the main robot body: (a) threaded insert nut installation, (b) SRF04 ultrasonic sensor installation, (c) DC socket installation.

In fundamental digital logic instruction, this training kit provides digital output indicators in the form of LEDs and a buzzer to represent high (1) and low (0) logic states. On the digital input side, various components are available, such as an ultrasonic sensor, push buttons, and a line sensor, all of which can be configured as binary logic signals. In addition, the training kit supports analog input learning in embedded control systems using a potentiometer, a DHT11 temperature sensor, and a line sensor that generates analog data. To enhance conceptual understanding and system implementation, the mobile robotic training kit is equipped with a TFT (Thin Film Transistor) LCD touchscreen, which functions to visualize data, display sensor information, and facilitate the development of graphical user interfaces (GUIs) in real-world applications, such as Internet of Things (IoT) systems and mobile robotic platforms. For actuation, DC motor control is supported by an L298N motor driver, enabling programmable control of motor direction and speed.

To provide a power source for the mobile robot training, the system is equipped with three 18,650 lithium batteries connected in series. It uses a Battery Management System (BMS) to protect the batteries from excessive voltage drops or surges, as well as from voltage spikes (Fig. 4). A single battery has a voltage of approximately 3.7 V; with three batteries connected in series, this produces a voltage of about 11.1 V. This voltage cannot be directly applied to sensors or other microcontroller inputs, which require a voltage range of 3.3 to 5 V; therefore, the training kit is equipped with an L7805 regulator IC to provide a 5-volt output. Thus, the training kit can provide two different voltage outputs: 5 V and approximately 12 V. The battery circuit designed in this training kit can be recharged continuously without needing to be removed from the kit. The configuration of the 5-volt regulator circuit used is shown in Fig. 3, and the battery circuit with BMS is shown in Fig. 4. In addition to using a battery, this training kit can also be operated with voltage directly from an external power supply via the DC socket (Fig. 4), where the power source can be selected via the selector switch shown in Fig. 2.

### 2.2 Mechanical design

Technically, the mobile robotic training kit measures 15 cm wide, 18 cm long, and 8 cm high, including the tallest component mounted on the platform. The frame of the training kit is fabricated from Polylactic Acid (PLA) using 3D printing technology. To construct the mechanical structure of the mobile robotic training kit, three main mechanical parts must be printed: the main body, the chassis, and the battery cover, as illustrated in Fig. 5. The main body of the robot (Fig. 5a) is designed to mount SRF04 ultrasonic sensors at the front, rear, left, and right sides, enabling the robot to detect the distance of surrounding objects from all four directions. This configuration enables the training kit to be implemented in wall-following robotic applications. In addition, a DC power socket is installed to recharge the battery or serve as an external power source for the training kit; its location is shown in Fig. 7.

Second, the robot chassis shown in Fig. 5(b) functions as the bottom cover of the training kit and as a mounting base for the 8-channel QTR-8RC line sensor used to detect color differences. This sensor is positioned on the underside of the chassis, as illustrated in Fig. 6, enabling it to detect black and white lines in line follower robot applications. In addition, the robot chassis serves as the housing for the battery, which acts as the primary power source, as shown in Fig. 6. To ensure the battery is securely installed and does not shift during operation, a specially designed battery cover is used, as shown in Fig. 5(c).

## 3 Design files summary

To manufacture the mobile robotic training kit, two primary design categories must be prepared: mechanical design (main robot body, robot chassis, and battery cover) and electronic design (robot electronic schematic and PCB board layout). In addition, a wiring guideline is required, as several components are not directly mounted on the main PCB, such as the line sensor, ultrasonic sensor, DC motors, battery module, and 12 V DC socket. A concise summary of all required files is presented in Table 1, which also includes a link to the website for accessing these resources. The mechanical design files can be fabricated using 3D printing, while the PCB board files can be produced independently if adequate equipment is available or through professional PCB fabrication services*.*

**Table 1:** Summary of all mobile robotic training kit design files.

Design file name	File type	Open source license	Location of the file
The main body of the robot	stp	Creative Commons Attribution 4.0 International	https://doi.org/10.5281/zenodo.20017448
Robot chassis	stp	Creative Commons Attribution 4.0 International	https://doi.org/10.5281/zenodo.20017448
Battery cover	stp	Creative Commons Attribution 4.0 International	https://doi.org/10.5281/zenodo.20017448
Robot electronic circuit	sch	Creative Commons Attribution 4.0 International	https://doi.org/10.5281/zenodo.20017448
Robot PCB board design	brd	Creative Commons Attribution 4.0 International	https://doi.org/10.5281/zenodo.20017448
Robot electrical circuit	cdr	Creative Commons Attribution 4.0 International	https://doi.org/10.5281/zenodo.20017448
Design after assembly	stp	Creative Commons Attribution 4.0 International	https://doi.org/10.5281/zenodo.20017448

## 4 Bill of materials summary

The components required to construct the mobile robotic training kit are categorized into two groups to facilitate clarity: electronic components (Table 2) and mechanical components (Table 3). For the mechanical components, all parts specified in Table 1 must also be fabricated. Regarding the electronic components, it should be noted that PCB fabrication was conducted with a minimum order quantity of five units, with a total cost of USD 67.70. Therefore, the PCB cost listed in the component table represents the estimated cost per board, calculated by dividing the total fabrication cost by the number of boards. Based on this estimate, the total cost of electronic components is approximately USD 55.09, while mechanical components cost approximately USD 18.93. Consequently, the total material cost for one unit of the training kit is approximately USD 74.02, excluding shipping expenses. It should be emphasized that these cost estimates may vary depending on the fabrication location and component pricing available to the researchers. Overall, the production cost of a single mobile robotic training kit is estimated at approximately USD 80. At this cost, educators receive a multifunctional training kit that can be used for embedded control system instruction at the fundamental level and for direct implementation in robotic applications. This cost-effectiveness constitutes a key advantage of the proposed training kit*.*

**Table 2:** Summary of electronic components of the mobile robotic training kit.

Designator	Component	N	Cost per unit ($)	Total cost ($)	Source of materials	Material type
PCB Board	Printing PCB Board	1	$13.54	$13.54	PCBWay	Composite
Electronic components	All electronic components of the mobile robotic training kit	1	$41.55	$41.55	Appendix 1	Other

Note: N = Number.

**Table 3:** Summary of mechanical components of the mobile robotic training kit.

Designator	Component	N	Cost per unit ($)	Total cost ($)	Source of materials	Material type
Filament	Mechanical Design 3D Printing Filament	1	$12.53	$12.53	Tokopedia	PLA
Bolt	Baut JP 3 × 15 mm + Nut M3	8	$0.03	$0.24	Tokopedia	Stainless steel
Bolt	Baut JP 3 × 20 mm + Nut M3	6	$0.04	$0.24	Tokopedia	Stainless stee
Bolt	Baut JP 2 × 6 mm + Nut M2	16	$0.09	$1.44	Tokopedia	Stainless stee
Bolt	Baut JP 2 × 20 mm + Nut M2	10	$0.10	$1.00	Tokopedia	Stainless stee
Nut	Insert Nut M3 × 4 × 3 mm	10	$0.04	$0.40	Tokopedia	Stainless stee
Bracket	Bracket Motor DC	4	$0.19	$0.76	Tokopedia	Plastic
Wheel	N20 Wheel	4	$0.58	$2.32	Tokopedia	Rubber

Note: N = Number, PLA = Polylactic Acid.

## 5 Build instructions

**Fig. 8:**
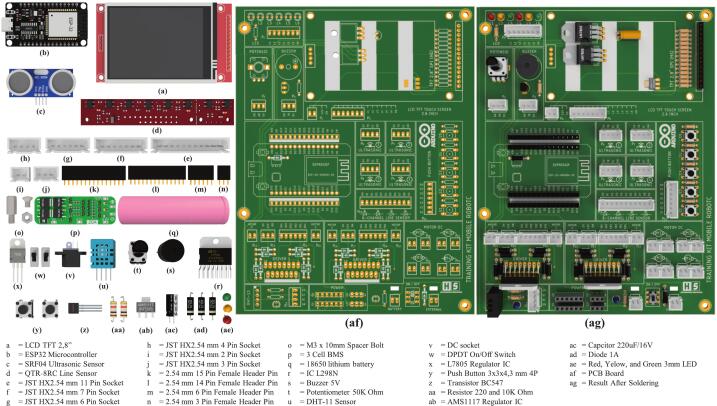
Installation of PCB components of the mobile robot.

Before initiating fabrication of the mobile robotic training kit, all required materials must be prepared, as listed in Table 2, Table 3. The mechanical components that must be produced using 3D printing technology are illustrated in Fig. 5, while the electronic components are clearly presented in Fig. 8. In this section, step-by-step instructions guide the assembly of the mobile robotic training kit. In addition, the complete manufacturing procedure is available in the learning module, which has been systematically developed, as summarized in Table 5.

### 5.1 Installation of robot chassis components

The robot chassis consists of three main components, as illustrated in Fig. 6. The installation process can be systematically carried out by following the steps outlined below:

1. First, install the 8-channel QTR-8RC line sensor from the underside of the chassis into the designated mounting slot and secure it using 3 × 15 mm screws, as shown in Fig. 6a.
2. Install the N20 DC motors onto the mounting brackets provided at the upper-right, lower-right, upper-left, and lower-left sections of the chassis. Secure each motor using 2 × 20 mm bolts. After all four motors are properly installed, attach the wheels to the N20 DC motors' shafts. This procedure is illustrated in Fig. 6b.
3. Install the lithium batteries in the central compartment of the robot chassis, which has been specifically designed to accommodate three lithium cells. After positioning the batteries, mount the Battery Management System (BMS) module above them. Finally, secure the assembly using the battery cover (Fig. 5c) and fasten it with 3 × 15 mm screws, as shown in Fig. 6c.

### 5.2 Installation of main robot body components

The main body of the robot also includes three components that must be installed separately: threaded insert nuts, ultrasonic sensors, and the DC socket, as illustrated in Fig. 7. The installation process can be systematically carried out by following the steps outlined below:

1. As shown in Fig. 7a, M3 × 4 × 3 mm threaded insert nuts are required for mounting the PCB and the lower chassis. The upper insert nuts are installed from the underside until fully seated in the designated screw holes, while the lower insert nuts are installed from the top until fully seated in the provided holes. It should be noted that the diameter of the mounting holes is intentionally smaller than that of the insert nuts to ensure a tight fit. Therefore, the insert nuts must be heated using a soldering iron and gently pressed into the holes until fully embedded. This procedure applies to both upper and lower insert nuts. Since the robot body is fabricated from PLA, special care must be taken during installation to prevent localized melting or deformation.
2. The SRF04 ultrasonic sensor is installed by inserting it from the inside of the body frame, facing outward, and securing it with 2 × 6 mm screws, as illustrated in Fig. 7b.
3. The DC socket is also installed on the inner side, facing outward, to allow an external DC jack to serve as the robot's power source. The socket is then fastened using 2 × 20 mm screws, as shown in Fig. 7c.

### 5.3 Soldering and installation of PCB board components

At this stage, three main steps must be completed to produce a PCB board ready for use in the mobile robotic training kit. These steps are outlined as follows:

1. Perform soldering of all PCB components onto the provided PCB board, as illustrated in Fig. 8af. Careful attention must be given to the correct placement of components, particularly polarized components such as capacitors and the L7805 voltage regulator IC. The L7805 regulator must be mounted in a horizontal (laid-down) position; otherwise, it may interfere with the LCD module that will later be installed above it. The component layout and the final soldering results are shown in Fig. 8ag.
2. Next, install the 2.8-inch TFT LCD onto the designated mounting supports and secure it using spacer bolts at the four mounting holes. The detailed installation procedure is illustrated in Fig. 9a.

**Fig. 9:**
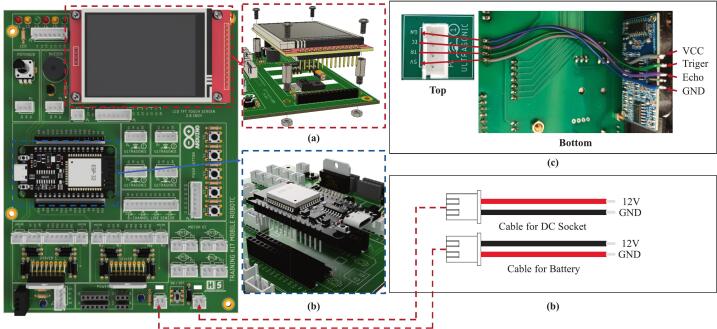
Installation of Components onto the Bracket: (a) LCD Installation, (b) Microcontroller Installation.
3. Finally, install the microcontroller onto the provided bracket using female header pins. The microcontroller can be mounted by directly attaching it to the female header connectors, as shown in Fig. 9b

### 5.4 Integration of all assembled main components

**Fig. 10:**
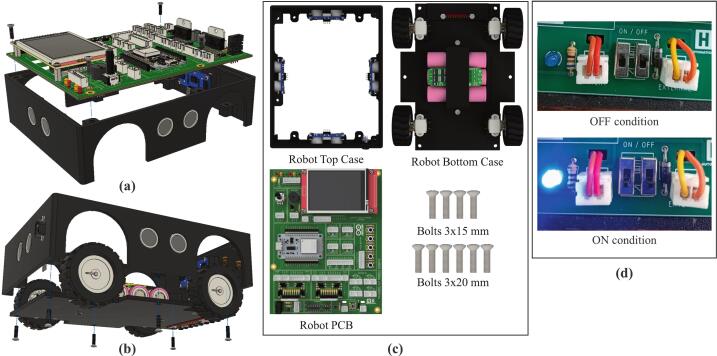
Assembly of all robot components: (a) pcb board installation, (b) robot chassis installation, (c) components to be prepared, (d) ON/OFF switch condition.

This process represents the final step in constructing the mobile robotic training kit. Before proceeding, ensure that the three main assembled components are fully prepared for integration, as illustrated in Fig. 10c. The steps to be followed in this stage are outlined as follows:

1. Attach the assembled PCB board to the main robot body, as illustrated in Fig. 10a, and secure it using 3 × 15 mm screws.
2. Perform the wiring installation by connecting the terminals on the PCB to components physically separated from the main board, including the line sensor, ultrasonic sensors, DC socket, DC motors, and battery module. The wiring configuration is illustrated in Fig. 4. To prevent errors during installation, careful attention must be given to the following steps:
a. First, connect the lithium batteries to the BMS module separately by referring to the wiring configuration shown in Fig. 4, without connecting them to the PCB board at this stage.
b. When connecting the ultrasonic sensor pins, ensure that each sensor pin is properly matched with the corresponding terminal label on the PCB board. As illustrated in Fig. 9c, the component terminals must be connected to the identically labeled pins on the upper side of the PCB. The installation of the ultrasonic sensors should follow the sensor numbering shown in Fig. 2a and align with the terminal numbering shown in Fig. 8ag.
c. The installation of the line sensor and DC motors follows the same principle as the ultrasonic sensors; careful attention must be paid to matching the PCB terminal labels with the corresponding component pins, in accordance with the wiring guide presented in Fig. 4.
d. Connect the battery and DC socket to the PCB using a 2-pin JST connector. In contrast, the opposite ends are soldered to the BMS input and the DC socket terminals through the designated holes on the PCB board, as illustrated in Fig. 9d. Before performing this step, ensure that the power switch is in the OFF position (Fig. 10d). This precaution prevents the robot from being immediately activated upon battery connection and minimizes the risk of damage due to wiring errors.
3. Carefully recheck all completed wiring installations to ensure the correctness and reliability of the assembled circuit.
4. After all installations are complete, as illustrated in Fig. 4, attach the robot chassis to the main robot body and secure it using 3 × 20 mm screws, as shown in Fig. 10b.

## 6 Operation instructions

Users of the mobile robotic training kit must comprehensively understand the operating procedures of both the hardware and software before conducting practicum activities. From the hardware perspective, primary attention should be given to the ON/OFF switch integrated into the robot. When the switch is in the OFF position, no power is distributed from the battery to the robot system, even if an external power source is connected; under this condition, the circuit functions solely for battery charging (Fig. 10d). Conversely, when the switch is in the ON position, power is supplied to the entire robot system from the battery. Connecting an external power source in this condition remains safe, as the system is equipped with a BMS that protects against overload, short circuit, and overcurrent and automatically terminates charging once the battery reaches full capacity (Fig. 10d). Operating the training kit with an external power source connected is recommended during fundamental practicum sessions to maintain a stable power supply. However, in the implementation of independent mobile robot projects (autonomous mobile robots), the external power source should be disconnected to prevent mechanical constraints that could affect system mobility and overall performance*.*

Second, users must comprehensively prepare the programming hardware and software environment using the Arduino IDE to ensure that the robot operates in accordance with the designed learning modules and job sheets. Before the training kit can be utilized optimally, several software components and system support packages must be installed as follows:

1. Install the Arduino IDE application on the computer or laptop to be used. The installation file can be downloaded from the official Arduino website, and the installation procedure may follow the tutorial provided in the following video: https://doi.org/10.5281/zenodo.20017448.
2. Install the CH340 driver, which functions as a communication interface between the computer and the ESP32 microcontroller board. Since the ESP32 board utilized in this study employs the CH340 communication IC, installing this driver is a prerequisite to ensure that program uploading and serial monitor communication operate properly. The driver file can be accessed via the following link: https://doi.org/10.5281/zenodo.20017448, and the installation procedure can be followed in this video tutorial: https://doi.org/10.5281/zenodo.20017448.
3. Add the ESP32 board support package to the Arduino IDE through the Boards Manager, as the Arduino IDE does not provide built-in support for ESP32 boards by default. Before initiating the installation process, users must first insert the following URL into the Arduino IDE Preferences menu as an additional board manager source: https://raw.githubusercontent.com/espressif/arduino-esp32/gh-pages/package_esp32_index.json. The installation procedure for the ESP32 board package in the Arduino IDE is covered in this video tutorial: https://doi.org/10.5281/zenodo.20017448.
4. Install the 2.8-inch TFT LCD library to enable optimal access and programming of graphical display features on the training kit. The installation procedure can follow this video tutorial: https://doi.org/10.5281/zenodo.20017448.
5. Install the DHT11 sensor library to enable temperature and humidity data acquisition in accordance with practicum requirements. The installation procedure can follow this video tutorial: https://doi.org/10.5281/zenodo.20017448.

All installation stages must be carried out systematically to ensure system compatibility, stable data communication, and the successful implementation of learning activities using the mobile robotic training kit. After users fully understand the hardware operating procedures and properly configure the required software environment, the training kit can be effectively utilized to support practicum activities. The researchers have developed an integrated series of practicum activities compiled within a single jobsheet, as presented in Table 5. Nevertheless, the utilization of this training kit is not limited to the provided guidelines, as users may further develop additional practicum scenarios aligned with the intended learning outcomes and targeted competencies.

## 7 Validation and characterization

**Fig. 11:**
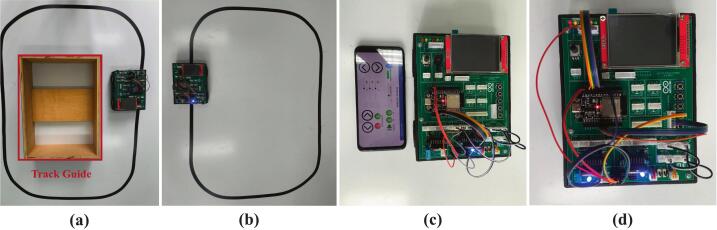
Application of the mobile robotic training kit: (a) wall follower robot, (b) line follower robot, (c) smartphone-controlled robot, (d) basic practicum.

**Fig. 12:**
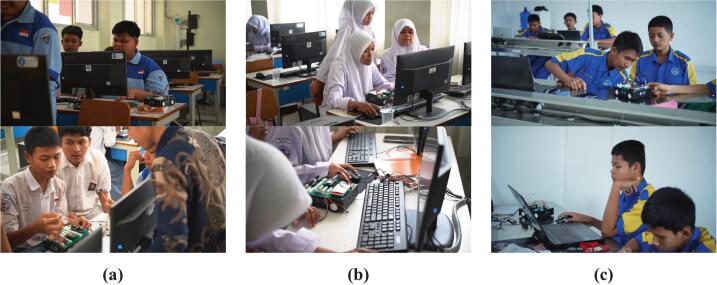
Implementation of the mobile robotic training kit in the learning process: (a) Vocational education A, (b) Vocational education B, (c) Vocational education C.

The training kit that was developed was implemented in the learning process with a broader range of participants, involving three vocational high schools in Sumatera Barat Province, Indonesia. For the purposes of this discussion, the researcher has replaced the names of the three schools with “Vocational Education A,” “B,” and “C” to maintain confidentiality and comply with the research code of ethics. The implementation involved 118 students and 6 teachers in Embedded Control Systems and spanned 16 meetings (one academic semester). The characteristics of all research samples (students and teachers) used in this study are presented in Table 4. The instructional process carried out by the six teachers was standardized using a curriculum and learning materials developed by the research team to ensure that the observed learning outcomes were attributable to the use of the training kit. The instructional materials, covering both foundational learning stages and project-based applications, are presented in Table 5. The practicum activities undertaken by students encompassed all features available in the third-generation mobile robotic training kit, ranging from fundamental exercises to real-world mobile robot applications, as illustrated in Fig. 11. Furthermore, the learning activities of students at each participating vocational institution are presented in Fig. 12*.*

**Table 4:** Characteristics of the research sample.

Characteristics	Group	Frequency	Percentage (%)
**Students**			
Gender	Male	83	70.34
	Female	35	29.66
Age	16 year	14	11.86
	17 – 18 years	96	81.36
	19 year	8	6.78
School	Vocational education A	46	38.98
	Vocational education B	41	34.75
	Vocational education C	31	26.27
Grade Level	XI (11th grade / Class 2)	118	100.00
**Teachers**			
Gender	Male	2	33.33
	Female	4	66.67
Age	30 to 40 years	2	33.33
	Above 40 years	4	66.67
School	Vocational education A	2	33.33
	Vocational education B	2	33.33
	Vocational education C	2	33.33

**Table 5:** Learning materials in the implementation of the mobile robotic training kit.

Learning Materials	Open source license	Location of the file
Learning Material Module	Creative Commons Attribution 4.0 International	https://doi.org/10.5281/zenodo.20017448
Practical Jobsheet	Creative Commons Attribution 4.0 International	https://doi.org/10.5281/zenodo.20017448

The mobile robotics training kit developed in this study is specifically designed for use by vocational high school students to understand the concepts and implementation of embedded control systems comprehensively. Based on the characteristics of the research sample, this device is recommended for 11th and 12th-grade students aged 16 to 19, who are cognitively at the formal operational stage and thus possess the ability to engage in abstract reasoning, systematic problem-solving, and the simultaneous integration of conceptual knowledge and practical skills [15], [26]. At this stage of development, students are not only able to understand the logic of microcontroller programming. However, they can also integrate it with the principles of electronic circuits and physical systems. Additionally, the use of this training kit requires prior foundational competencies, including a basic understanding of electronics—such as component identification, circuit analysis, and the principles of sensors and actuators—as well as soldering skills, which are typically acquired during earlier vocational education phases (10th grade). These foundational competencies serve as an essential foundation in supporting students’ success in designing, assembling, and functionally implementing microcontroller-based control systems [18], [27]. It is these foundational competencies that students must master to address the challenges encountered during the assembly process of the mobile robotic training kit. Once assembly is complete, students can learn the basic concepts of embedded control systems through the application of mobile robotics.

In the default configuration, 14 lab topics are designed in a step-by-step manner to help students apply concepts in circuits and microcontroller programming. The lab topics are as follows:

1. Concepts of Digital Input and Output Programming in Microcontrollers aims to provide an understanding of the basic concepts of digital input and output circuits in microcontrollers. In this training, a mobile robotics training kit is used, employing a push button as a digital input and an LED as a digital output. As a result, students are expected to be able to use all types of digital inputs, including sensors and mechanical inputs, as well as microcontroller output devices.
2. Concept of Analog Input Programming for Microcontrollers aims to understand the basic concepts of analog input circuits and programming for microcontrollers. In the lab session, a training kit is used with a simulator to process analog values from a potentiometer. Through this lab, students are also expected to use all types of analog input devices, including sensors and mechanical components.
3. Pulse Width Modulation (PWM) Output Programming Concept, aimed at understanding the basic concepts of PWM circuits and programming for microcontrollers, used for controlling motor speed, light brightness, and other applications utilizing microcontroller PWM output. In the lab, a potentiometer and a push button are used to adjust the PWM value, while an LED serves as a visual representation of the PWM output.
4. Branching Programming Concept, aimed at understanding the basic logic concepts of microcontroller programming for decision-making. Two basic components are used: a push button and an LED.
5. DC Motor Control Concept, aimed at understanding the circuit and programming concepts for controlling the speed and direction of a DC motor using a motor driver. There are three basic components used in this lab: a push button as an input, a motor driver, and a DC motor.
6. 0.8 “TFT LCD Touchscreen Programming Concept, aimed at understanding the basic circuit concepts for communication between a TFT LCD and a microcontroller, as well as the basic programming for creating text and fill patterns as display elements (such as buttons or indicators) on the LCD interface. Thus, the only component used here is a 2.8″ TFT LCD.
7. Programming Concept for Buttons and Indicator Lights on a 2.8 “TFT LCD Touchscreen, aims to understand the programming concepts for button and indicator light interfaces on a TFT LCD. The components used in this lab are a 2.8″ TFT LCD and LEDs.
8. Concept of Programming Numerical Data for a 2.8″ TFT LCD Touchscreen aims to understand the programming concept of transmitting numerical data as values from a microcontroller output, such as PWM values or other values that can be sent from the LCD to the microcontroller. Two components are used in this lab: a TFT LCD and an LED, with the LCD displaying the LED's PWM value.
9. Programming Concept of the HC-SRF04 Ultrasonic Sensor aims to understand the circuitry and programming of the HC-SRF04 ultrasonic sensor as a distance measurer. Thus, in the training kit, the HC-SRF04 sensor and TFT LCD are used to display the measurement results.
10. Programming Concept of the QTR-8RC Line Sensor aims to understand the circuit and programming concepts of the line sensor, which operates on analog data to distinguish between two colors: black and white. Thus, two components are used: the QTR-8RC sensor and a TFT LCD to display the sensor's readings.
11. Bluetooth ESP32 Communication Concept to Smartphone aims to understand the concept of wireless communication programming via Bluetooth using the ESP32 microcontroller. Two components are used: a motor driver and a DC motor, which respond to data sent via Bluetooth. Meanwhile, the data sent is displayed using serial monitoring in this lab session.
12. Bluetooth-Controlled Robot Project: This is the first project undertaken by students, and in terms of difficulty, it is the easiest for students to complete. There are two basic components in the training kit: a motor driver and a DC motor, which are controlled via a smartphone. To conduct this lab, students must first master five prerequisite labs: Labs 1, 3, 4, 5, and 11.
13. Line Follower Robot Project is the second project undertaken by students, with a difficulty level considered intermediate in the learning process. The objective is for students to learn how to design a microcontroller-based embedded control system in robotics applications. To complete this lab, students must first master five prerequisite labs: Labs 1, 3, 4, 5, and 10.
14. The Wall Follower Robot Project is the final project undertaken by the students and is considered the most challenging in the course. The objective is the same: to teach students how to design microcontroller-based embedded control systems for robotics applications. To undertake this lab, students must first master five prerequisite labs: 1, 3, 4, 5, and 9.

This series of laboratory exercises is systematically structured from the conceptual level to project-based implementation, so that students not only understand the basic principles of microcontroller programming but also can integrate sensors, actuators, display interfaces, and wireless communication into a complete robotic system. This step-by-step approach is intended to strengthen conceptual understanding, technical skills, and problem-solving abilities in the development of microcontroller-based control systems. For more details on the lab steps, circuits, experimental programs, and learning exercises, please refer to the lab worksheet in Table 5.

**Fig. 13:**
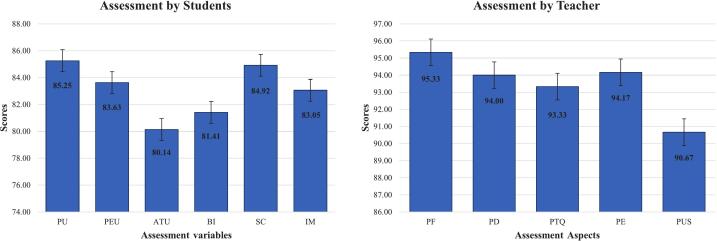
Results of the evaluation by students and teachers.

After one semester of implementation, the developed training kit was evaluated by students using the Technology Acceptance Model (TAM). The evaluation was conducted through a questionnaire survey to measure the level of acceptance of the training kit in embedded systems learning. The assessment instrument was based on standardized indicators developed by Chatzopoulos et al. [28] and Chen et al. [29], encompassing four primary constructs: perceived usefulness (PU), perceived ease of use (PEU), attitude toward using (ATU), and behavioral intention (BI). In addition to the TAM constructs, this study also assessed students’ self-confidence and intrinsic motivation during the learning process. Self-confidence (SC) was measured because confidence in academic ability has been shown to significantly influence active engagement, self-regulation, and learning success in the use of technology-based instructional media [30]. Meanwhile, intrinsic motivation (IM) was analyzed due to its critical role in fostering learning persistence, problem-solving ability, and meaningful learning without reliance on external incentives [31]. The evaluation results of the mobile robotic training kit are presented in Fig. 13.

Based on the quantitative evaluation using the TAM enriched with affective constructs, the PU score was 85.25—the highest among all indicators—indicating that students strongly perceived the mobile robotic training kit as providing tangible benefits in supporting embedded systems and robotics learning [32]. The SC score was also high, approximately 84.92, suggesting that the use of the training kit effectively enhanced students’ confidence in completing technology-based practicum and projects [26]. Meanwhile, PEU and IM scored around 83.63 and 83.05, respectively, reflecting that the training kit was relatively easy to use and capable of fostering students’ intrinsic learning motivation [33]. However, the scores for ATU and BI were comparatively lower, approximately 80.14 and 81.41, respectively. This indicates that although students recognized the usefulness, ease of use, and positive impacts on confidence and motivation, their affective attitudes and intentions to continue using similar technology had not yet fully developed optimally.

In addition to student evaluations, the mobile robotic training kit was also assessed by teachers as users in the embedded systems learning process. The evaluation was conducted using a questionnaire covering five variables: product functions (PF), product design (PD), product technical quality (PTQ), product ergonomics (PE), and product use safety (PSU) [34], [35], [36]. The evaluation results are presented in Fig. 13. Based on the assessment of the training kit’s quality, PF received the highest score of 95.33, indicating that the functions provided by the training kit fully met the needs of embedded systems, microcontroller, and robotics learning, and effectively supported practicum and project activities. PD and PE also achieved relatively high scores, approximately 94.00 and 94.17, respectively, suggesting that the physical design and component layout of the training kit were considered attractive, easy to operate, and comfortable for students during the learning process. Meanwhile, PTQ scored around 93.33, reflecting that the technical performance, system reliability, and hardware–software integration were rated as good, although there is room for further improvement. On the other hand, PSU received the lowest score of 90.67, indicating that usage safety remains a key concern, particularly regarding component protection, electrical safety, and risk mitigation during practicum. Nonetheless, overall evaluations showed high scores, all above 90, demonstrating strong positive feedback from teacher users.

Based on the research findings, the multifunctional mobile robotic training kit developed and implemented for the teaching of embedded systems, microcontrollers, and robotics was well received and utilized in the learning process, as evidenced by student and teacher evaluations. However, the effectiveness of this approach on student learning outcomes compared to other instructional media remains unknown. The evaluation results indicate that the third-generation training kit provides significant educational benefits in the learning process, as evidenced by the high PU, PEU, SC, and IM scores of the students, suggesting that this medium is not only user-friendly but also fosters self-confidence and intrinsic motivation in completing technology-based practical exercises and projects. On the other hand, teachers’ assessments of product quality revealed excellent performance in terms of functionality, design, technical quality, ergonomics, and safety of use, confirming that the training kit meets vocational learning needs both functionally and pedagogically. Overall, these findings confirm that the developed mobile robotic training kit has the potential to serve as an innovative learning medium relevant to the needs of vocational education and the demands of technology-based industries.

Furthermore, learning supported by this mobile robotic training kit contributes to strengthening students' achievement of the Work Competency Standards in the fields of prototype electronics and programming, as officially established by the Ministry of Manpower of the Republic of Indonesia as a reference for competency training and certification [37]. The implementation of this training kit specifically facilitates mastery of several key competency units namely 7 out of the total 44 required competency units including: basic microcontroller embedded system programming, control system-based embedded system programming, microcontroller-based wireless serial communication, visual interface development on mobile or desktop devices, IoT-based microcontroller programming, integrated cloud database development, and cloud-based user interface design for IoT systems. Support for these competency units is made possible by the training kit's integration of essential components, such as sensors and actuators, which enable students to understand basic programming concepts and implement control systems in the context of mobile robotics. Additionally, the use of the ESP32 microcontroller, equipped with a wireless communication module and a TFT display, provides students with the opportunity to develop IoT systems, implement wireless data communication, and integrate with cloud services. Mastery of these competencies, reinforced through a certification scheme, opens career opportunities in electronics engineering, industrial automation, and microcontroller-based robotics. However, given the context of implementation in vocational secondary education, the competencies developed remain at the basic to intermediate level, serving as a foundation for more advanced professional competencies.

Based on the evaluation results, the mobile robotic training kit demonstrated good performance in supporting the learning process; however, several limitations must be explicitly acknowledged. First, the complexity of the assembly process, which involves the integration of mechanical, electronic, and programming aspects, requires a relatively long time and adequate technical skills, which poses a challenge for users with limited experience. Second, implementing learning using this training kit tends to be more effective in small groups (2–3 students), as it can improve work efficiency, collaboration, and minimize technical errors, particularly among students with limited initial competencies. Third, the design of this training kit focuses on mastering basic to intermediate concepts in embedded control systems, so it does not yet fully accommodate learning needs at the advanced level. Therefore, further development has been considered in subsequent research, particularly through enhancing learning content and the complexity of the control system. Currently, the control approach used is still conventional, such as basic if–else logic; in the future, the integration of more complex control methods will be developed, namely the Proportional Integral Derivative (PID) control system, which is widely used in industrial applications due to its ability to improve the precision and stability of control systems [38], [39]. It is hoped that this development will improve the alignment between the competencies acquired by students in the vocational education learning process and the competency requirements in the industrial world.

## Ethics statements

This research has been approved by the principals of three vocational high schools in Sumatera Barat Province, Indonesia, as the research sites, and has been approved by the Head of the Sumatera Barat Provincial Education Office via letter No. 000.9/5921/PSMK/DISDIK-2025*.*

## CRediT authorship contribution statement

**Herlin Setyawan:** Writing – original draft, Software, Methodology, Investigation, Conceptualization. **Sukardi:** Supervision, Conceptualization, Methodology, and Data curation. **Risfendra:** Software, Visualization, Validation, and Data curation. **Habibullah:** Data curation and Writing – review & editing. **Ganefri:** Writing – review & editing. **Muhammad Anwar:** Writing – review & editing.

## Declaration of competing interest

The authors declare that they have no known competing financial interests or personal relationships that could have appeared to influence the work reported in this paper.

## Appendix A Supplementary data

Supplementary Data 1 Components List of Mobile Robot Training Kit.

Supplementary Data 2 Assessment Instruments by Students.

Supplementary Data 3 Assessment Instruments by Teachers.

Supplementary Data 4 Main Repository for Storing All Mobile Robotics Training Kit Assembly Files.
