# Supplementary material for: Development and implementation of a multifunctional mobile robot training kit in embedded control systems instruction in vocational education
Source: HardwareX. 2026 May 11;26:e00789. doi: 10.1016/j.ohx.2026.e00789 (PMC13199891; doi:10.1016/j.ohx.2026.e00789)
Supplement: Supplementary Data 1 — Components List of Mobile Robot Training Kit. [file mmc1.docx]

**Appendix 1**

**Components List of Mobile Robot Training Kit**

**Electronic Components List of Mobile Robot Training Kit**

| **No** | **Designator** | **Component** | **N** | **Cost per unit ($)** | **Total cost ($)** | **Source of materials** | **Material type** |
| --- | --- | --- | --- | --- | --- | --- | --- |
| 1 | PCB Board | Printing PCB Board | 1 | $13.54 | $13.54 | [PCBWay](https://www.pcbway.com/orderonline.aspx) | Composite |
| 2 | LCD | LCD TFT 2,8” Touch Screen | 1 | $6.92 | $6.92 | [Tokopedia](https://www.tokopedia.com/syalis-electrical/display-touch-tft-lcd-oled-2-8-inch-arduino-14-pin-syalis) | Semiconductor |
| 3 | Microcontroller | ESP32 Microcontroller | 1 | $4.19 | $4.19 | [Tokopedia](https://www.tokopedia.com/rajacell/esp-32-new-development-board-usb-type-c-esp32-wroom-wifi-bluetooth-30pinch340c-409b0) | Semiconductor |
| 4 | Ultrasonic Sensor | SRF04 Ultrasonic Sensor | 4 | $0.75 | $3.00 | [Tokopedia](https://www.tokopedia.com/cncstorebandung/hc-sr04-ultrasonic-distance-measuring-sensor-transducer-3-3v-5v-untuk-arduino-esp32-diy-elektronika-proyek-iot-1731127919069333054?extParam=src%3Dshop%26whid%3D15512&aff_unique_id=&channel=others&chain_key=) | Semiconductor |
| 5 | Line Sensor | QTR-8RC Line Sensor | 1 | $1.61 | $1.61 | [Tokopedia](https://www.tokopedia.com/cncstorebandung/sensor-line-tracking-line-follower-robot-8-channel?extParam=src%3Dshop%26whid%3D15512&aff_unique_id=&channel=others&chain_key=) | Semiconductor |
| 6 | Pin Connector | JST HX2.54 mm 11 Socket | 1 | $0.44 | $0.44 | [Tokopedia](https://www.tokopedia.com/s3-satriasecure/molex-mini-11pin-2-54-molek-2-54mm-header-11-pin-jst-xh-2-54-mm-soket-terminal-11p-11-pin-konektor-connector-11-p-kaki-11pin-konector-conektor-11kaki-11lubang-11-lubang-jalur-11jalur-1731323195457177380) | Stainless steel |
| 7 | Pin Connector | JST HX2.54 mm 7 Socket | 3 | $0.08 | $0.23 | [Tokopedia](https://www.tokopedia.com/latronika/soket-molek-kecil-7-pin-set-terminal-socket-molex-konektor-xh2-54-7p) | Stainless steel |
| 8 | Pin Connector | JST HX2.54 mm 6 Socket | 2 | $0.07 | $0.13 | [Tokopedia](https://www.tokopedia.com/latronika/soket-molek-kecil-6-pin-set-terminal-socket-molex-konektor-xh2-54-6p) | Stainless steel |
| 9 | Pin Connector | JST HX2.54 mm 4 Socket | 5 | $0.22 | $1.10 | [Tokopedia](https://www.tokopedia.com/s3-satriasecure/molex-mini-4pin-2-54-molek-2-54mm-header-4-pin-jst-xh-2-54-mm-soket-4p-terminal-4p-4-pin-konektor-connector-4-p-kaki-4pin-konector-conektor-4kaki-4lubang-4-lubang-jalur-4jalur-1731323187788547876?extParam=src%3Dshop%26whid%3D311029&aff_unique_id=&channel=others&chain_key=) | Stainless steel |
| 10 | Pin Connector | JST HX2.54 mm 2 Socket | 15 | $0.17 | $2.55 | [Tokopedia](https://www.tokopedia.com/s3-satriasecure/molex-mini-2pin-2-54-molek-2-54mm-header-2-pin-jst-xh-2-54-mm-soket-2p-konektor-terminal-kabel-2p-2-pin-connector-2-p-kaki-2pin-konector-conektor-2kaki-2lubang-2-lubang-jalur-2jalur-1731323187873548068?extParam=src%3Dshop%26whid%3D311029&aff_unique_id=&channel=others&chain_key=) | Stainless steel |
| 11 | Pin Connector | JST HX2.54 mm 3 Socket | 4 | $0.20 | $0.80 | [Tokopedia](https://www.tokopedia.com/s3-satriasecure/molex-mini-3pin-2-54-molek-2-54mm-header-3-pin-jst-xh-2-54-mm-soket-3pin-konektor-terminal-kabel-3p-3-pin-connector-3-p-kaki-3pin-konector-conektor-3kaki-3lubang-3-lubang-jalur-3jalur-1731323207987857188?extParam=src%3Dshop%26whid%3D311029&aff_unique_id=&channel=others&chain_key=) | Stainless steel |
| 12 | Pin Connector | 2.54 mm 15 Pin Female Header | 2 | $0.11 | $0.22 | [Tokopedia](https://www.tokopedia.com/efst/pin-header-female-betina-row-pcb-1x40-2-54mm-1729604684576164015?extParam=whid%3D3957701%26src%3Dshop&aff_unique_id=&channel=others&chain_key=) | Stainless steel |
| 13 | Pin Connector | 2.54 mm 14 Pin Female Header | 1 | $0.11 | $0.11 | [Tokopedia](https://www.tokopedia.com/efst/pin-header-female-betina-row-pcb-1x40-2-54mm-1729604684576164015?extParam=whid%3D3957701%26src%3Dshop&aff_unique_id=&channel=others&chain_key=) | Stainless steel |
| 14 | Pin Connector | 2.54 mm 6 Pin Female Header | 2 | $0.03 | $0.06 | [Tokopedia](https://www.tokopedia.com/rajacell/female-header-1x3-1x4-1x5-1x6-1x8-1x10-1x6-straight-pin-lurus-vertikal-pitch-2-54mm-1732329720427546564?extParam=src%3Dshop%26whid%3D328226&aff_unique_id=&channel=others&chain_key=) | Stainless steel |
| 15 | Pin Connector | 2.54 mm 3 Pin Female Header | 2 | $0.02 | $0.05 | [Tokopedia](https://www.tokopedia.com/rajacell/female-header-1x3-1x4-1x5-1x6-1x8-1x10-1x6-straight-pin-lurus-vertikal-pitch-2-54mm-1732329720427349956?extParam=src%3Dshop%26whid%3D328226&aff_unique_id=&channel=others&chain_key=) | Stainless steel |
| 16 | Pin Connector | Male Jumper Pins | 40 | $0.02 | $0.80 | [Tokopedia](https://www.tokopedia.com/aisyahrobot/1set-male-black-housing-1p-dupont-jumper-wire-pin-connector-2-54mm-1730890362895893926?extParam=whid%3D1351425%26src%3Dshop&aff_unique_id=&channel=others&chain_key=) | Stainless steel |
| 17 | Spacer Bolt | M3 x 10mm Spacer Bolt | 4 | $0.09 | $0.36 | [Tokopedia](https://www.tokopedia.com/efst/spacer-10mm-1cm-m3-kaki-dudukan-pcb-kuningan-metal-besi-1-set-hex-aluminium?extParam=src%3Dshop%26whid%3D3957701&aff_unique_id=&channel=others&chain_key=) | Stainless steel |
| 18 | Power supply | 3 Cell BMS | 1 | $0.60 | $0.60 | [Tokopedia](https://www.tokopedia.com/snapshot_product?dtl_id=3368270532&order_id=1630006727) | Semiconductor |
| 19 | Power supply | 18650 lithium battery | 3 | $1.15 | $3.45 | [Tokopedia](https://www.tokopedia.com/cncstorebandung/baterai-batre-battery-18650-rill-1500-mah?extParam=src%3Dshop%26whid%3D15512&aff_unique_id=&channel=others&chain_key=) | Lithium |
| 20 | Power supply | L7805 Regulator IC | 2 | $0.10 | $0.20 | [Tokopedia](https://www.tokopedia.com/cncstorebandung/cnc-l7805cv-l7805-7805-to-220-positive-voltage-regulator-5v?extParam=src%3Dshop%26whid%3D15512&aff_unique_id=&channel=others&chain_key=) | Semiconductor |
| 21 | Power supply | AMS1117 Regulator IC | 1 | $0.05 | $0.05 | [Tokopedia](https://www.tokopedia.com/rajacell/ams1117-33v-ldo-voltage-regulator-sot-223-smd-ams-1117-33v) | Semiconductor |
| 22 | Power supply | 12V 2A DC Adapter | 1 | $1.16 | $1.16 | [Tokopedia](https://www.tokopedia.com/efst/adaptor-dc-12v-2a-5-5mm-12-v-volt-2-a-ampere-psu-power-supply-trafo-1729596168070138031?extParam=src%3Dshop%26whid%3D3957701&aff_unique_id=&channel=others&chain_key=) | Semiconductor |
| 23 | Buzzer | Buzzer 5V | 1 | $0.10 | $0.10 | [Tokopedia](https://www.tokopedia.com/cncstorebandung/buzzer-speaker-active-3v-5v-12x9-5mm-modul-alarm-suara-mini-for-arduino-esp32-uno-mega-nano-diy-elektronik-project-1733103146066150974?extParam=src%3Dshop%26whid%3D15512&aff_unique_id=&channel=others&chain_key=) | Semiconductor |
| 24 | Potentiometer | Potentiometer 50K Ohm | 1 | $0.10 | $0.10 | [Tokopedia](https://www.tokopedia.com/rajacell/rv09-0932-potensiometer-analog-potentiometer-10k-20k-50k-100k-50k-vertical) | Carbon |
| 25 | Temperature sensor | DHT-11 Sensor | 1 | $0.77 | $0.77 | [Tokopedia](https://www.tokopedia.com/rajacell/dht11-dht-11-sensor-temperature-humidity-tanpa-breakout-board-normal) | Semiconductor |
| 26 | Socket | DC Socket | 1 | $0.08 | $0.08 | [Tokopedia](https://www.tokopedia.com/s3-satriasecure/jack-soket-dc-kuping-socket-female-sasis-kontra-power-in-ada-lubang-screw-baut-adaptor-cewe-konektor-terminal-kabel-adapter-audio-video-1731323201968965412?extParam=src%3Dshop%26whid%3D311029&aff_unique_id=&channel=others&chain_key=) | Stainless steel |
| 27 | Switch | DPDT On/Off Switch | 2 | $0.06 | $0.11 | [Tokopedia](https://www.tokopedia.com/efst/slide-switch-spdt-1p2t-saklar-geser-kecil-mini-toggle-3-pin-pcb-4mm-1729607449770690735?extParam=ivf%3Dfalse%26keyword%3Dswitch+dpdt+kecil%26search_id%3D20260203083250320722549D1751261CUO%26src%3Dsearch) | Stainless steel |
| 28 | Push Button | Push Button 3x3x4,3 mm 4P | 5 | $0.03 | $0.15 | [Tokopedia](https://www.tokopedia.com/cncstorebandung/cnc-tactile-switch-push-button-6x5x5mm?extParam=src%3Dshop%26whid%3D15512&aff_unique_id=&channel=others&chain_key=) | Stainless steel |
| 29 | Transistor | Transistor BC547 | 1 | $0.12 | $0.12 | [Tokopedia](https://www.tokopedia.com/s3-satriasecure/bc548-bc548-transistor-bc-548-renceng-rencengan-rentet-rentetan-bc-548-part-aksesoris-audio-video-dan-lain-lain-1731323201934427940) | Semiconductor |
| 30 | Resistor | Resistor 220 Ohm | 3 | $0.01 | $0.03 | [Tokopedia](https://www.tokopedia.com/cncstorebandung/10x-resistor-1-4w-1-metal-film-value-1k-10k-220-330-4k7-10-100-470-ohm-untuk-arduino-elektronika-diy-1732355104787695166?extParam=src%3Dshop%26whid%3D15512&aff_unique_id=&channel=others&chain_key=) | Carbon |
| 31 | Resistor | Resistor 670 Ohm | 1 | $0.01 | $0.01 | [Tokopedia](https://www.tokopedia.com/cncstorebandung/10x-resistor-1-4w-1-metal-film-value-1k-10k-220-330-4k7-10-100-470-ohm-untuk-arduino-elektronika-diy-1732355104788022846?extParam=src%3Dshop%26whid%3D15512&aff_unique_id=&channel=others&chain_key=) | Carbon |
| 32 | Resistor | Resistor 10K Ohm | 6 | $0.01 | $0.05 | [Tokopedia](https://www.tokopedia.com/cncstorebandung/10x-resistor-1-4w-1-metal-film-value-1k-10k-220-330-4k7-10-100-470-ohm-untuk-arduino-elektronika-diy-1732355104787629630?extParam=src%3Dshop%26whid%3D15512&aff_unique_id=&channel=others&chain_key=) | Carbon |
| 33 | IC Driver Motor | IC L298N | 2 | $1.06 | $2.12 | [Tokopedia](https://www.tokopedia.com/rajacell/ic-l298n-zip-15-chip-motor-driver-full-dual-channel-h-bridge) | Semiconductor |
| 34 | Capacitor | Capacitor 220uF/16V | 1 | $0.03 | $0.03 | [Tokopedia](https://www.tokopedia.com/putraniagabdg/elco-220uf-16v-kapasitor-electrolit-polar-220-uf-16-volt-105?extParam=ivf%3Dfalse%26keyword%3Dcapacitor+220uf%2F16volt%26search_id%3D2026020307572911B9842E0A271F246UYR%26src%3Dsearch) | Electrolyte |
| 35 | Diode | Diode 1A | 18 | $0.01 | $0.16 | [Tokopedia](https://www.tokopedia.com/cncstorebandung/dioda-penyearah-1n4007-1a-1000v-10pcs-1n4001-1a-50v-10pcs-rectifier-diode-do-41-untuk-power-supply-rangkaian-elektronik-1733628484460447294?extParam=src%3Dshop%26whid%3D15512&aff_unique_id=&channel=others&chain_key=) | Semiconductor |
| 36 | LED | Red LED | 2 | $0.01 | $0.02 | [Tokopedia](https://www.tokopedia.com/rajacell/led-3mm-diffused-red-merah-lampu-led-diode-high-quality) | Semiconductor |
| 37 | LED | Yellow LED | 2 | $0.01 | $0.02 | [Tokopedia](https://www.tokopedia.com/rajacell/led-3mm-diffused-yellow-kuning-lampu-led-diode-high-quality) | Semiconductor |
| 38 | LED | Green LED | 2 | $0.01 | $0.02 | [Tokopedia](https://www.tokopedia.com/rajacell/led-3mm-diffused-green-hijau-lampu-led-diode-high-quality) | Semiconductor |
| 39 | LED | Blue LED | 2 | $0.01 | $0.02 | [Tokopedia](https://www.tokopedia.com/selchel/led-3mm-diffused-merah-kuning-hijau-putih-biru-orange-6313a?extParam=ivf%3Dfalse%26keyword%3Dled+3mm+diffused+biru+merah%26search_id%3D20260203075602577BCC914F9E55169YO2%26src%3Dsearch) | Semiconductor |
| 40 | USB Cable | USB to Type C Cable | 1 | $0.83 | $0.83 | [Tokopedia](https://www.tokopedia.com/efst/kabel-data-charger-usb-type-c-rotate-putar-180-fast-charge-cable-tebal-1-meter-led-uslion?extParam=src%3Dshop%26whid%3D3957701&aff_unique_id=&channel=others&chain_key=) | Copper |
| 41 | Stranded Cable | 16p Rainbow Ribbon Cable | 1 | $0.71 | $0.71 | [Tokopedia](https://www.tokopedia.com/efst/kabel-pita-pelangi-16p-16-p-pin-jalur-rainbow-ribbon-cable-idc-strip-1729602209680099503?extParam=src%3Dshop%26whid%3D3957701&aff_unique_id=&channel=others&chain_key=) | Copper |
| 42 | Motor DC | Motor DC N20 12V | 4 | $2.02 | $8.08 | [Tokopedia](https://www.tokopedia.com/cncstorebandung/motor-dc-ga12-n20-micro-dc-gear-motor-motor-gearbox-torsi-tinggi-untuk-diy-robotik-1731127927925278270?extParam=ivf%3Dfalse%26keyword%3Ddc+micro+motor+n30%26search_id%3D202602030931478D3BBA7425E7C91CB3S6%26src%3Dsearch) | Metal |
| **Total** | | | |  | **$55.09** |  |  |

**Mechanical Components List of Mobile Robot Training Kit**

| **No** | **Designator** | **Component** | **N** | **Cost per unit ($)** | **Total cost ($)** | **Source of materials** | **Material type** |
| --- | --- | --- | --- | --- | --- | --- | --- |
|  | Filament | Mechanical design 3D printing filament | 1 | $0.03 | $0.24 | [Tokopedia](https://www.tokopedia.com/ftoday/esun-3d-printer-filament-pla-1-75mm-1kg-original-hitam?extParam=ivf%3Dfalse%26keyword%3Dfilament+esun%26search_id%3D20260203095805320722549D17512E13TL%26src%3Dsearch) | PLA |
|  | Bolt | Baut JP 3 x 15 mm + Nut M3 | 8 | $0.04 | $0.24 | [Tokopedia](https://www.tokopedia.com/armcomp/baut-3x15mm-3x20mm-mur-baut-jp-3x15-3x20-m3-15mm-20mm-25mm-30mm-15mm?extParam=ivf%3Dfalse%26keyword%3Dbaut+jp+3+x+20+mm+%2B+nut+m3%26search_id%3D20260203095349CD00692C3BFE592382CX%26src%3Dsearch) | Stainless steel |
|  | Bolt | Baut JP 3 x 20 mm + Nut M3 | 6 | $0.09 | $1.44 | [Tokopedia](https://www.tokopedia.com/armcomp/baut-3x15mm-3x20mm-mur-baut-jp-3x15-3x20-m3-15mm-20mm-25mm-30mm-20mm?extParam=ivf%3Dfalse%26keyword%3Dbaut+jp+3+x+20+mm+%2B+nut+m3%26search_id%3D20260203095349CD00692C3BFE592382CX%26src%3Dsearch) | Stainless stee |
|  | Bolt | Baut JP 2 x 6 mm + Nut M2 | 16 | $0.10 | $1.00 | [Tokopedia](https://www.tokopedia.com/archive-lapaktukang/1set-baut-mur-ring-jp-stainless-m2x6-m2x8-m2x10-m2x12-m2x14-m2x16-m2x20-m2-m2x6-mm-baut-mur-ring-3717b?extParam=ivf%3Dfalse%26keyword%3Dbaut+jp+2+x+20+mm+%2B+nut+m2%26search_id%3D20260203095148CAF1A3DFB505FF183GMO%26src%3Dsearch) | Stainless stee |
|  | Bolt | Baut JP 2 x 20 mm + Nut M2 | 10 | $0.04 | $0.40 | [Tokopedia](https://www.tokopedia.com/archive-lapaktukang/1set-baut-mur-ring-jp-stainless-m2x6-m2x8-m2x10-m2x12-m2x14-m2x16-m2x20-m2-m2x20-mm-baut-mur-ring-21853?extParam=ivf%3Dfalse%26keyword%3Dbaut+jp+2+x+20+mm+%2B+nut+m2%26search_id%3D20260203095148CAF1A3DFB505FF183GMO%26src%3Dsearch) | Stainless stee |
|  | Nut | Insert Nut M3 x 4 x 3 mm | 10 | $0.19 | $0.76 | [Tokopedia](https://www.tokopedia.com/powerpop/brass-knur-m2-m3-insert-nut-mur-tanam-3d-print-m3-x-4-x-3) | Stainless stee |
|  | Bracket | Bracket motor DC | 4 | $0.58 | $2.32 | [Tokopedia](https://www.tokopedia.com/jogjarobotika/bracket-motor-dc-gearbox-n20-holder-mur-baut-putih?extParam=ivf%3Dfalse%26keyword%3Dbracket+motor+n20%26search_id%3D20260203094914F9B902FC3289AF336ZNU%26src%3Dsearch&t_id=1770112161421&t_st=1&t_pp=search_result&t_efo=search_pure_goods_card&t_ef=goods_search&t_sm=&t_spt=search_result) | Plastic |
|  | Wheel | N20 Wheel | 4 | $0.03 | $0.24 | [Tokopedia](https://www.tokopedia.com/ichibot/roda-ban-motor-dc-n20-wheel-ukuran-43mm-karet?extParam=ivf%3Dfalse%26keyword%3Droda+n20%26search_id%3D2026020309502094F6D7E04A4D450420P1%26src%3Dsearch) | Rubber |
| **Total** | | | |  | **$18.93** |  |  |
| **Grand total** | | | |  | **$74.02** |  |  |
