# Supplementary material for: Development and implementation of a multifunctional mobile robot training kit in embedded control systems instruction in vocational education
Source: HardwareX. 2026 May 11;26:e00789. doi: 10.1016/j.ohx.2026.e00789 (PMC13199891; doi:10.1016/j.ohx.2026.e00789)
Supplement: Supplementary Data 2 — Assessment Instruments by Students. [file mmc2.docx]

**Appendix 2**

**Assessment Instruments by Teachers**

**Notes:**

The evaluation was conducted with 118 students (N = 118) from three vocational education institutions in Indonesia, namely SMK Negeri 1 Sumatera Barat, SMK Negeri 5 Padang, and SMK Pembangunan Teknologi Robotik UNP. Each assessment item was rated on a five-point Likert scale from 1 to 5. The mean (M) and standard deviation (SD) values obtained for each assessment item are also reported. The values ​​listed are still in the range of 1 to 5 and have not been converted to values ​​of 0 to 100 as shown in the research results.

| **No** | **Statement** | **M** | **SD** |
| --- | --- | --- | --- |
| **Perceived Usefulness (PU) (M = 4.26, SD = 0.66)** | | | |
| PU1 | Using the training kit allows me to complete tasks more quickly | 4.12 | 0.67 |
| PU2 | Using the training kit enhances my knowledge | 4.12 | 0.68 |
| PU3 | Using the training kit improves my performance | 4.20 | 0.66 |
| PU4 | Using the training kit increases my learning autonomy | 4.56 | 0.55 |
| PU5 | The mobile robotic training kit is useful for learning embedded systems | 4.31 | 0.66 |
| **Perceived Ease of Use (PEU) (M = 4.18, SD = 0.68)** | | | |
| PEU1 | Using the training kit facilitates my learning process | 4.38 | 0.64 |
| PEU2 | The mobile robotic training kit is easy to learn | 4.12 | 0.67 |
| PEU3 | I find that using the training kit is not complicated and does not require much mental effort | 4.12 | 0.68 |
| PEU4 | The training kit is flexible for use in conducting practical activities | 4.20 | 0.66 |
| PEU5 | I can easily become proficient in using the mobile robotic training kit | 4.08 | 0.72 |
| **Attitude Toward Using (ATU) (M = 4.01, SD = 0.74)** | | | |
| ATU1 | I enjoy using the mobile robotic training kit in the learning process | 3.97 | 0.70 |
| ATU2 | Using the training kit in learning activities increases my confidence in learning | 3.98 | 0.76 |
| ATU3 | Using the mobile robotic training kit in learning is a good idea | 3.96 | 0.77 |
| ATU4 | Using the mobile robotic training kit in learning is a wise idea | 4.10 | 0.74 |
| ATU5 | I like the idea of using the mobile robotic training kit in learning | 4.03 | 0.73 |
| **Behavioral Intention (BI) (M = 4.07, SD = 0.64)** | | | |
| BI1 | I plan to use the mobile robotic training kit regularly during this semester | 3.99 | 0.67 |
| BI2 | I plan to use the mobile robotic training kit for basic practical activities and experiments in the learning process | 4.11 | 0.62 |
| BI3 | I intend to frequently use the mobile robotic training kit in the future | 4.11 | 0.63 |
| **Self-Confidence (SC) (M = 4.25, SD = 0.74)** | | | |
| SC1 | I am confident that I can work effectively on a wide range of tasks | 4.42 | 0.67 |
| SC2 | When faced with difficult tasks, I am confident that I can complete them successfully | 4.14 | 0.82 |
| SC3 | I believe that I can succeed in almost all endeavors that I undertake | 4.18 | 0.68 |
| **Intrinsic Motivation (IM) (M = 4.15, SD = 0.67)** | | | |
| IM1 | In learning activities using the mobile robotic training kit, I prefer activities that stimulate my curiosity, even if they are challenging to learn | 4.23 | 0.63 |
| IM2 | In learning activities using the mobile robotic training kit, I am more interested in challenging tasks that allow me to learn new things | 4.15 | 0.67 |
| IM3 | The most satisfying aspect of using the mobile robotic training kit is when I am able to thoroughly understand how the system works | 3.93 | 0.77 |
| IM4 | I enjoy the learning process using the mobile robotic training kit in the classroom | 4.30 | 0.56 |
