# Supplementary material for: Development and implementation of a multifunctional mobile robot training kit in embedded control systems instruction in vocational education
Source: HardwareX. 2026 May 11;26:e00789. doi: 10.1016/j.ohx.2026.e00789 (PMC13199891; doi:10.1016/j.ohx.2026.e00789)
Supplement: Supplementary Data 3 — Assessment Instruments by Teachers. [file mmc3.docx]

**Appendix 3**

**Assessment Instruments by Teachers**

**Notes:**

The assessment was carried out by 6 embedded systems subject teachers (N=6) using a Likert scale with 5-point responses. The following also shows the mean (M) value and standard deviation (SD) for each assessment item. The values ​​listed are still in the range of 1 to 5 and have not been converted to values ​​of 0 to 100 as shown in the research results.

| **No** | **Statement** | **M** | **SD** |
| --- | --- | --- | --- |
| **Product Functions (PF) (M = 4.77, SD = 0.50)** | | | |
| PF1 | The training kit functions effectively in accordance with the intended learning objectives | 4.83 | 0.41 |
| PF2 | The components of the training kit operate reliably and stably | 4.67 | 0.82 |
| PF3 | The use of the training kit assists students in understanding embedded systems concepts and programming | 4.83 | 0.41 |
| PF4 | The training kit effectively supports basic practical activities | 4.83 | 0.41 |
| PF5 | The training kit effectively supports experimental activities in embedded systems | 4.67 | 0.52 |
| **Product Design (PD) (M = 4.70, SD = 0.53)** | | | |
| PD1 | The physical design of the training kit attracts learners’ attention | 4.50 | 0.55 |
| PD2 | The component layout is well organized | 5.00 | 0.00 |
| PD3 | Component labels or markings assist users during the learning process | 4.67 | 0.82 |
| PD4 | The user interface of the mobile robotic training kit is easy to understand | 4.50 | 0.55 |
| PD5 | The design of the training kit supports ease of maintenance | 4.83 | 0.41 |
| **Product Technical Quality (PTQ) (M = 4.67, SD = 0.47)** | | | |
| PTQ1 | The components of the training kit comply with educational standards | 4.83 | 0.41 |
| PTQ2 | The components of the training kit are of good quality | 4.67 | 0.52 |
| PTQ3 | The electrical system of the training kit exhibits stable power performance during operation | 4.50 | 0.55 |
| PTQ4 | The frame structure and mechanical materials demonstrate adequate physical durability for repeated use | 4.67 | 0.52 |
| PTQ5 | The technical documentation provides comprehensive information, including schematics, connection diagrams, component specifications, and user-friendly maintenance guidelines | 4.67 | 0.52 |
| **Product Ergonomics (PE) (M = 4.71, SD = 0.45)** | | | |
| PE1 | The training kit is easy to use in the learning process | 4.67 | 0.52 |
| PE2 | The placement of buttons, ports, and components is easily accessible | 4.83 | 0.41 |
| PE3 | The user guide or operating instructions are clear and easy to follow | 4.67 | 0.52 |
| PE4 | The use of the training kit helps reduce instructional time | 4.67 | 0.52 |
| **Product Use Safety (PUS) (M = 4.53, SD = 0.81)** | | | |
| PUS1 | The training kit is safe to use in classroom learning activities | 4.83 | 0.41 |
| PUS2 | The components are protected against risks such as short circuits or reverse polarity | 4.33 | 0.52 |
| PUS3 | User errors do not result in serious system damage | 4.33 | 1.21 |
| PUS4 | There are no parts that pose potential safety hazards | 4.33 | 1.21 |
| PUS5 | The training kit is equipped with safety features such as fuses or current protection mechanisms | 4.83 | 0.41 |
