# Supplementary material for: Development and implementation of a multifunctional mobile robot training kit in embedded control systems instruction in vocational education
Source: HardwareX. 2026 May 11;26:e00789. doi: 10.1016/j.ohx.2026.e00789 (PMC13199891; doi:10.1016/j.ohx.2026.e00789)
Supplement: Supplementary Data 4 — Main Repository for Storing All Mobile Robotics Training Kit Assembly Files. [file mmc4.docx]

Main Repository for Storing All Mobile Robotics Training Kit Assembly Files

<https://doi.org/10.5281/zenodo.20017448>
